# Long-term clinical results of alumina ceramic medial pivot total knee arthroplasty: a 10-year follow-up study

**DOI:** 10.1186/s42836-023-00180-9

**Published:** 2023-05-19

**Authors:** Hideki Ueyama, Shigeru Nakagawa, Yuichi Kishimura, Yukihide Minoda, Suguru Nakamura, Junichiro Koyanagi, Mitsuyoshi Yamamura, Yoshinori Kadoya

**Affiliations:** 1 Department of Orthopedic Surgery, Osaka Rosai Hospital, 1179-3 Nagasonecho, Kita Ward, Sakai, Osaka 591-8025 Japan; 2 Department of Orthopedic Surgery, Hanwa Joint Reconstruction Center, 3176 Hukaikitamachi Naka Ward, Sakai, Osaka 599-8271 Japan; 3 Department of Orthopedic Surgery, Osaka Metropolitan University Graduate School of Medicine, 1-4-3 Asahimachi, Abeno, Osaka 545-8585 Japan

**Keywords:** TKA, Medial pivot, Ceramic, Long-term result, Survival rate, Longevity

## Abstract

**Background:**

The newly-designed alumina ceramic medial pivot total knee prosthesis was introduced to reduce polyethylene wear and better fit the anatomical morphology of the Asian population. This study aimed to clarify the long-term clinical results of alumina medial pivot total knee arthroplasty over a minimum follow-up period of 10 years.

**Methods:**

The data of 135 consecutive patients who underwent primary alumina medial pivot total knee arthroplasty were analyzed in this retrospective cohort study. Patients were examined over a minimum 10-year follow-up period. The knee range of motion, Knee Society Score (KSS) knee score, Knee Society Score function score, and radiological parameters were assessed. The survival rate was also evaluated by using reoperation and revision as endpoints.

**Results:**

The mean follow-up period lasted 11.8 ± 1.4 years. Patients who were not followed accounted for 7.4% of the total cohort. Knee and function scores of KSS improved significantly following total knee arthroplasty (*P* < 0.001). In 27 individuals (28.1%), a radiolucent line was observed. Aseptic loosening occurred in three cases (3.1%). The survival rates for reoperation and revision were 94.8% and 95.8% 10 years after the operation, respectively.

**Conclusions:**

During a minimum 10-year follow-up period, the present model of alumina medial pivot total knee arthroplasty showed good clinical outcomes and survival rates.

## Background

Total knee arthroplasty (TKA) is a successful surgery for degenerative knee diseases with good long-term results. Many TKA prostheses are made of metals, such as cobalt-chrome (CoCr). However, prostheses made of alumina ceramic have also been reported to impart good longevity [1]. Ceramic implants reportedly demonstrated fewer intra-articular polyethylene (PE) particles than their metal counterparts [2], which could reduce aseptic loosening in the long term. Nonetheless, unlike metals, ceramics possess an evident drawback of possible fracture resulting from mechanical force [3]. It is important to observe the long-term survivorship of each prosthetic model since the possibility of implant failure increases with time following TKA [4].

**Fig. 1:**
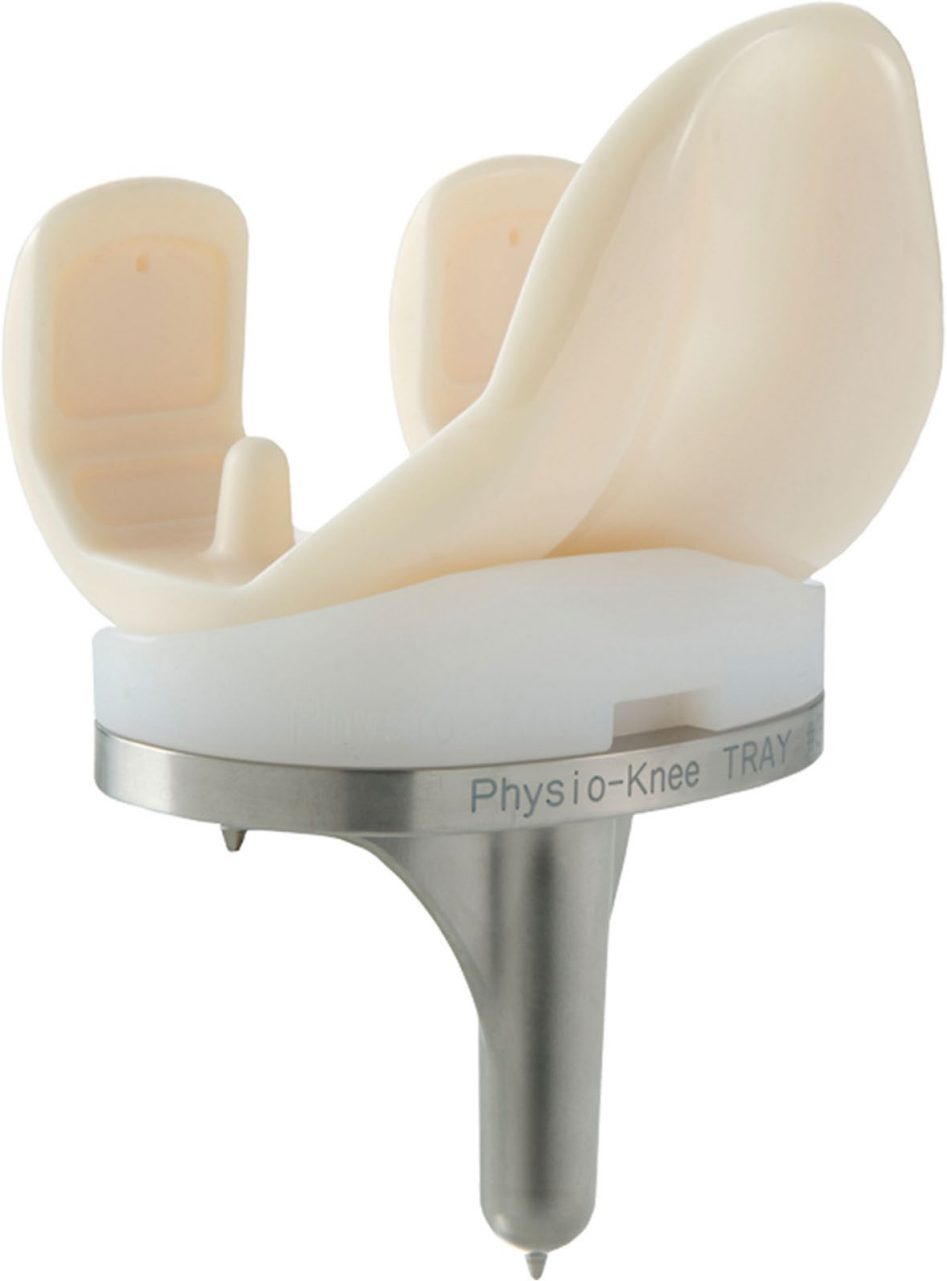
The current alumina medial pivot total knee prosthesis model (Physio-knee system^®^; Kyocera Corporation, Kyoto, Japan), comprising a ball-and-socket joint and less-congruent articulation medially and laterally, respectively. The femoral and tibial components are composed of alumina ceramic and Ti_6_Al_4_V, respectively

Medial pivot TKA has demonstrated good long-term survivorship. Nonetheless, it has been noted that radiolucent lines (RLLs) tend to appear around the anterior flange of the femoral component [5]. Furthermore, medial pivot total knee prostheses made of alumina ceramic also required revision following aseptic loosening of the femoral component [1]. Finite element analysis demonstrated tensile stress to be concentrated at the anterior flange of the femoral component during activities of daily living [6]. A large femoral component or excessive posterior condyle could cause flexion gap tightness, and the flexion tightness could increase the mechanical force on the femoral component [7]. The current model of alumina medial pivot total knee prosthesis (Physio-knee system®, Kyocera, Kyoto, Japan), which has a larger femoral mediolateral/anteroposterior (ML/AP) ratio to better fit the bone morphology of East Asians [8], might have a favorable effect on the long-term survivorship (Fig. 1).

This study aimed to investigate the clinical results of alumina medial pivot TKA using the current model over a minimum 10-year follow-up period. The hypothesis was that the current model would yield good clinical results and survivorship.

## Methods

### Study design

Included in this retrospective cohort study were consecutive patients who underwent TKA using the Physio-knee system^®^ for knee osteoarthritis (OA) at our institution from January 2007 to July 2011. The exclusion criteria were the presence of knee diseases other than OA and the inability to visit the hospital. The data were analyzed after a minimum 10-year follow-up period.

### Surgical technique

All TKAs were performed by a single surgical team using an identical surgical procedure. The team consisted of four senior surgeons who were all trained as knee joint specialists and had more than 10 years of experience. After resection of the posterior cruciate ligament, the knee joint was exposed via a medial parapatellar approach. The femoral and tibial bones were resected by using the gap technique, and bone cement was utilized to fix the prostheses. The patellae were resurfaced for any cases. In every case, cruciate substitution PE inserts were applied. Based on the institution's therapeutic protocols for TKA, postoperative pain management and physical therapy were provided [5].

### Outcomes

The Knee Society Score (KSS) and range of motion (ROM) were assessed preoperatively and at the last follow-up examination [9]. According to a prior study, the knee ROM was assessed using a conventional clinical goniometer [10]. Short film radiographs of the standing knee taken preoperatively and at the end of the follow-up period were used to evaluate radiological parameters. Postoperative alignments of components (*α*, *β*, *γ*, *δ*) [11] and femorotibial angles before and after surgery were assessed [12]. RLLs were evaluated, and loosening was defined as the presence of RLLs ≥ 2 mm or changes in prosthetic alignment [13]. Radiological measurements were performed by two physicians unrelated to TKA. Twenty patients were measured twice with a 6-week interval to calculate the intraclass correlation coefficient (ICC) and validate the measurement method [14]. Reoperation referred to any additional surgery following TKA, such as exchange of insert, debridement and irrigation, or revision. In contrast, the revision was considered to be surgery performed to remove one or more component(s) for any reason [5].

### Statistical analysis

Continuous variables were presented as means and standard deviations (SD), while categorical variables were expressed as absolute frequencies. The same patients were compared before and after TKA using univariate analysis, with the paired *t*-test used for continuous variables and Fisher’s exact test for categorical data. During the follow-up period, the survival rate was calculated using the Kaplan–Meier analysis, the endpoints being re-operation and revision, with a 95% confidence range [15]. The risk factors for revision and reoperation were identified by multivariate logistic regression analysis. Based on a prior study, a power analysis was performed to establish the right number of patients required to detect a difference of approximately 15° ± 15° in the ROM or a difference of 30 ± 15 points in the KSS [1, 16]. A two-sided alpha value of 0.01 and more than 24 examples were needed to achieve a statistical power of 80%. Statistical significance was defined as a *P*-value < 0.01. Statistical analyses were performed using the R software package (version 3.1.1; R Core Team 2014. R Foundation for Statistical Computing, Vienna, Austria).

## Results

**Fig. 2:**
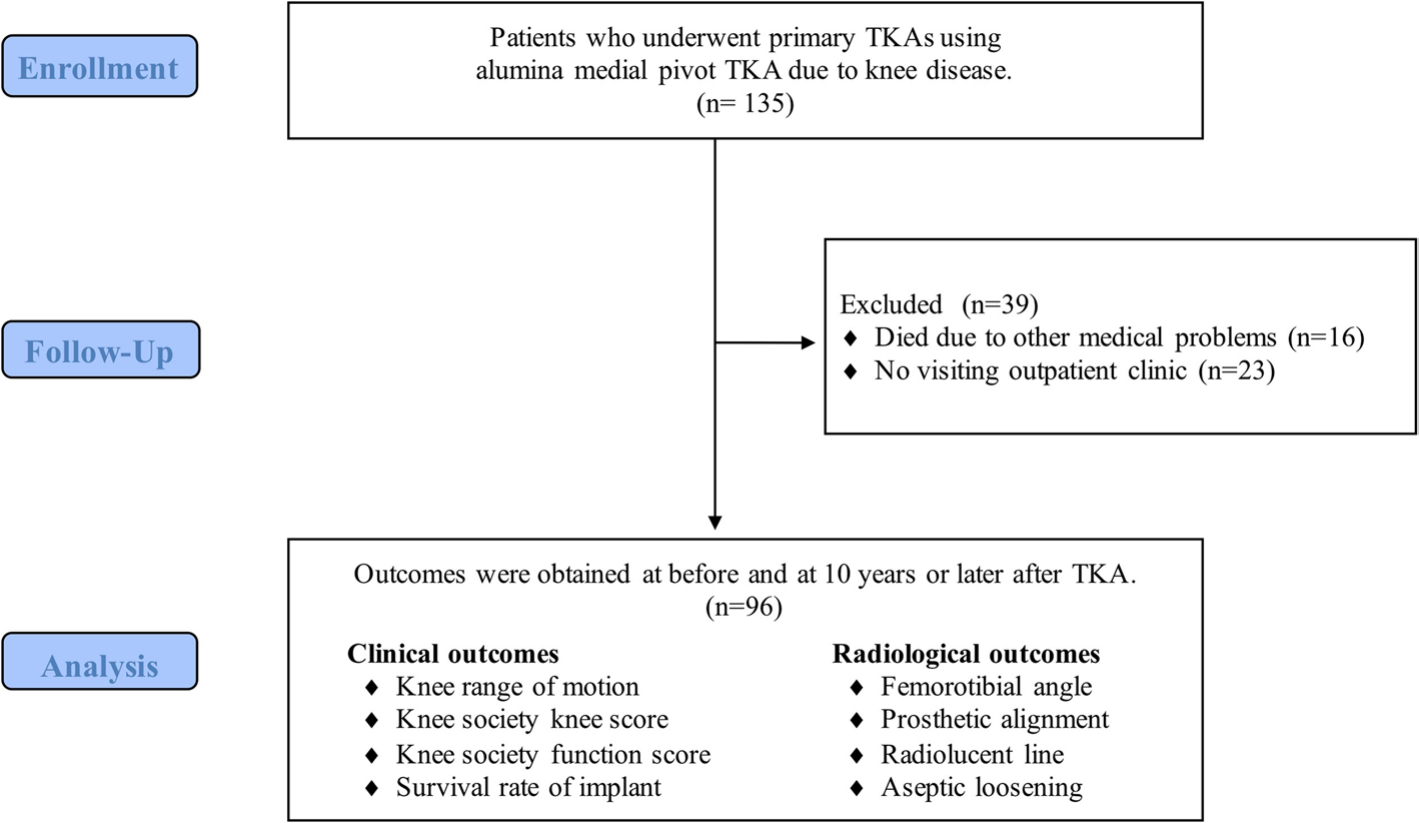
The flow diagram of this single-arm observational study. Patients who underwent total knee arthroplasty were followed for a minimum of 10 years

**Fig. 3:**
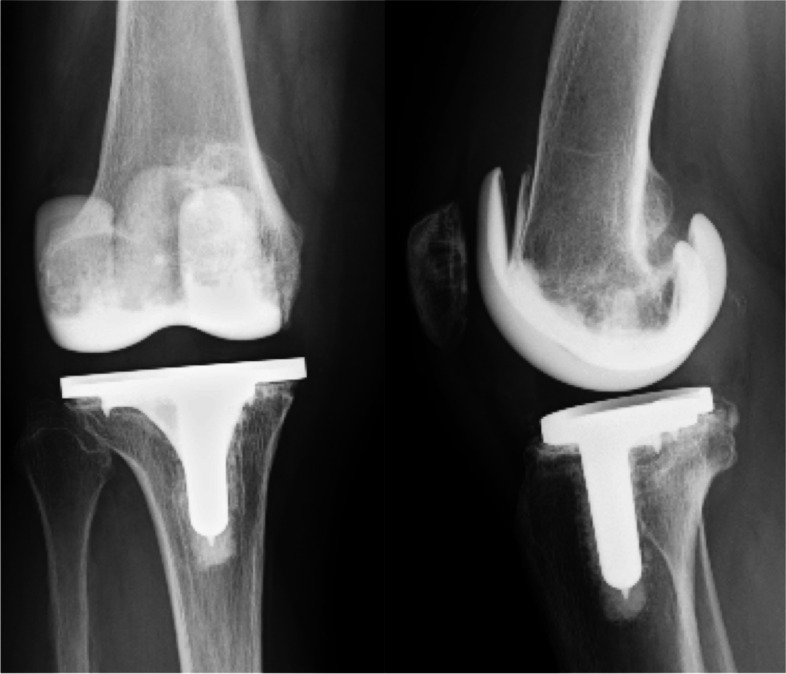
Radiological imaging of aseptic loosening requiring femoral and tibial component replacement. At 5 years postoperatively, radiolucency ≥ 2 mm and a radiolucent line < 2 mm appeared at the anterior flange of the femoral component and the tibial component, respectively

**Fig. 4:**
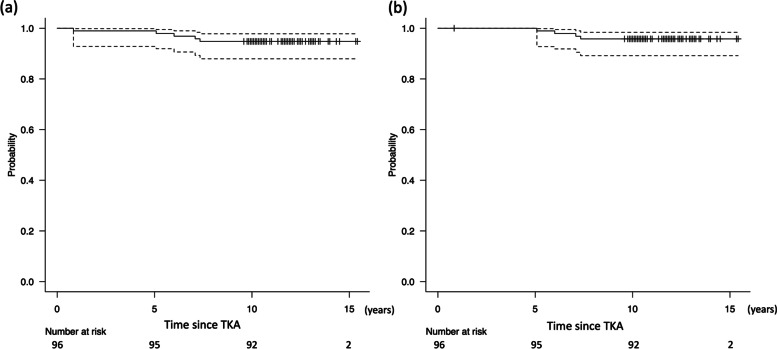
Kaplan–Meier survivorship of alumina medial pivot prostheses to re-operation (**a**) and revision (**b**) with 95% confidence intervals (dashed lines). Tick marks indicate censored patients. The survival rates were 94.8% and 95.8% for reoperation and revision at 10 years after procedure

In total, 135 consecutive patients were enrolled in this study. Sixteen patients died of medical issues unrelated to the knee disease, 13 patients were unable to visit the outpatient clinic for various reasons, such as relocation (these patients confirmed by telephone that the knee condition was functionally adequate for daily living), and 10 patients could not be followed up. These patients were defined as being lost to follow-up and were excluded from the study. Finally, the data of 96 patients who were followed up for a minimum period of 10 (mean, 11.8 ± 1.4) years were analyzed (Fig. 2). It should be noted that 7.4% of patients were lost to follow-up. Table 1 summarizes the demographic characteristics of the patients. In Table 2, the clinical and radiological parameters prior to TKA and at the final follow-up examination are presented. The KSS knee score, function score, knee extension, and femorotibial angle were significantly improved at the final follow-up examination. Intra-observer ICC was 0.72 (95% confidence interval (CI): 0.40–0.87) and the inter-observer ICC was 0.69 (95% CI: 0.34–0.85). Based on a previous report, both measurement reliabilities can be defined as “good” [17]. RLLs were observed in 27 patients (28.1%). RLLs appeared in 27 and 2 cases at the anterior and posterior areas of the femoral component, respectively, and in 16, 1, 4, and 1 case(s) in the medial, lateral, anterior, and posterior areas of the tibial component, respectively. In some cases, RLLs appeared in multiple areas. Aseptic loosening occurred in two cases in the femoral component and in one case in the tibial component (Fig. 3). Five reoperations and four revisions took place over the minimum 10-year follow-up (Table 3). The survival rates at 10 years following TKA, with reoperation and revision as the endpoints, were 94.8% and 95.8% (Fig. 4, Table 4). No specific risk factors for aseptic loosening of the components were detected (Table 5).

**Table 1:** Patient demographics

**Parameters**	***n* = 96**
Age at operation (years)	70.2 ± 7.4
Woman (*n*, %)	90 (94%)
Height (cm)	150.2 ± 5.9
Weight (kg)	61.3 ± 10.2
BMI (kg/m^2^)	27.2 ± 4.2
Varus deformity of the knee (*n*, %)	96 (100%)
Osteoarthritis (*n*, %)	96 (100%)
Follow-up period (years)	11.8 ± 1.4

Mean and standard deviation were provided. Number and percentage were also provided

*BMI* Body mass index

**Table 2:** Clinical and radiological parameters at pre-operation and final follow-up

**Parameters**	**Preoperation**	**Final follow-up**	***P*-value**
	***n* = 96**	***n* = 96**	
Clinical outcomes			
KSS knee score (points)	44.1 ± 4.9	89.9 ± 7.8	< 0.001
KSS function score (points)	45.5 ± 4.9	83.8 ± 7.6	< 0.001
Knee extension (°)	-5.4° ± 6.8°	-0.8° ± 2.9°	< 0.001
Knee flexion (°)	120° ± 13°	116° ± 14°	0.36
Radiological outcomes			
Femorotibial angle (°)	183° ± 9.4°	176° ± 3.0°	< 0.001
Prosthetic alignment (°)			
*α*	-	94.2° ± 1.8°	N.A
*β*	-	90.7° ± 1.7°	N.A
*γ*	-	2.5° ± 3.2°	N.A
*δ*	-	3.3° ± 4.0°	N.A
Radiolucent line (*n*, %)	-	27 (28.1%)	N.A
Aseptic loosening (*n*, %)	-	3 (3.1%)	N.A

Mean and standard deviation were provided. Number and percentage were also provided. *P*-values were calculated between preoperation and final follow-up

*KSS* Knee Society Score, *N.A.* Not applicable

**Table 3:** Details of re-operation and treatment

**Reasons for re-operation**	***n* (%)**	**Treatment *(n*, %)**
Aseptic loosening	3 (3.1%)	Revision of total components (3, 3.1%)
Septic loosening	1 (1.0%)	Two-stage revision of total components (1, 1.0%)
Acute surgical site infection	1 (1.0%)	Debridement and irrigation, and the exchange of polyethylene insert (1, 1.0%)
Total of re-operation	5 (5.2%)	All of above
Total of revision	4 (4.2%)	Revisions of above

The rate of each parameter was calculated from the total cases (*n* = 96)

**Table 4:** Summary of survival rates

**Time (years)**	**Number at risk**	**Survival rate (%)**	**95% CI**
Re-operation for any reasons			
1	96	99.0%	92.8–99.9
5	95	97.9%	91.9–99.5
6	94	96.9%	90.6–99.0
7	93	95.8%	89.3–98.4
8	92	94.8%	87.9–97.8
10	92	94.8%	87.9–97.8
Revision for any reasons			
5	95	98.9%	92.8–99.9
6	94	97.9%	91.8–99.5
7	93	96.8%	90.5–99.0
8	92	95.8%	89.2–98.4
10	92	95.8%	89.2–98.4

*CI* Confidence interval

**Table 5:** Multiple logistic regression model for risk factors of aseptic loosening

Predictors	Odd’s ratio (95% CI)	*P*-value
Age at operation	0.89 (0.72–1.10)	0.25
Sex (woman)	< 0.001 (N.A.)	0.99
Operation side (right knee)	4.50 (0.34–59.8)	0.25
BMI	0.94 (0.70–1.26)	0.69
Preoperative knee extension	0.79 (0.59–1.06)	0.11
Preoperative knee flexion	0.92 (0.83–1.03)	0.15
Preoperative femorotibial angle	0.91 (0.64–1.31)	0.61
Bone resection (measured technique)	0.49 (0.02–13.6)	0.62
Operation time	1.00 (0.91–1.11)	0.96
Postoperative prosthetic alignments		
*α*	0.91 (0.44–1.85)	0.79
*β*	0.77 (0.34–1.55)	0.47
*δ*	0.93 (0.54–1.57)	0.78
*γ*	1.05 (0.98–1.12)	0.16

*P-*value of this model was 0.03. A total of 96 cases were estimated in this model

*BMI* Body mass index, *CI* Confidence interval, *N.A.* Not applicable

## Discussion

This study demonstrated that the current alumina medial pivot total knee prosthesis model produced good clinical results without serious complications due to its good prosthesis design, and its survivorship was 95.8% over the minimum 10-year follow-up period. This survivorship was comparable to that of many other models used for TKA worldwide [18].

A long-term observation showed that aseptic loosening caused by PE wear particles is one of the most common causes of revision TKA [19]. Ceramics is harder and the surface is less rough than CoCr, which is commonly used in TKA implants [20, 21]. A hip simulator study on PE wear between ceramic-on-PE and metal-on-PE bearing surfaces demonstrated that ceramic-on-PE bearing surfaces reduced wear by 50% [22]. Therefore, although the phenomenon of PE wear should be interpreted with caution because the tribology of TKA and THA is different, in theory, alumina TKA exerts a favorable effect on PE wear. Intra-articular PE wear particles following TKA were less in previous alumina medial pivot total knee prosthesis models than in other metal prostheses in vivo [2, 23]. These features of alumina total knee prostheses, which have the potential to reduce wear-related failures, may have resulted in the good long-term survivorship of the current model in this study.

Previous alumina total knee prosthesis models have been reported to impart good longevity with a 10-year survivorship being 99% [1]. However, as the cited study excluded all patients who could not be followed up for 10 years after TKA, the population size for survivorship analysis was smaller than that of this study and the result was affected by bias. Therefore, the survival rate of this previous model should be interpreted with caution. The previous model had a larger ML/AP ratio than the current one, rendering it unsuitable for the anatomical features of patients of East Asian origin [8]. The design of the current model improved the fit by considering the anatomical features of the Asian population. Therefore, it could be more compatible, thereby ensuring a more surgeon-friendly approach. Furthermore, there is a learning curve to keep in mind when considering the results of a new model. For newly-introduced models, clinical results may not stabilize until the surgeon exceeds the learning curve [24, 25]. However, the cases analyzed in this study were registered 18 months after the introduction of the models to the domestic market, when all surgeons were fully familiar with this model. Therefore, this study could specifically evaluate the clinical performance of this model.

Ceramic TKA implants may also be a good option for patients with metal allergies. It is not easy to found out and decide whether adverse reactions are caused by the implanted metal material [23, 26, 27]. Therefore, eliminating possible causative agents would be safer when performing TKA for patients with metal allergies. Nickel is the most common agent attributed to metal allergy [22, 23]. Therefore, alumina TKA, which does not contain nickel, is safe and easy for patients who are suspected to have a metal allergy.

Many reports have demonstrated that medial pivot TKA is associated with good patient satisfaction and prosthesis longevity. However, these studies only evaluated metal implants [5, 13, 24–26]. As the risks specific to ceramics were not reported in these studies, the complications of alumina medial pivot TKA require clarification. An inherent risk of ceramics is that they can fracture upon forceful impact [3]. Although this risk was pointed out in a previous report [28], no case of ceramic fracture occurred in vivo from surgery to the final follow-up examination in this study. However, as the impact force may result in ceramic fracture, surgeons should be cautious in using a hammer during surgery. On the basis of the results of our study, the safety of alumina TKA model is believed to be comparable with that of common metal implants.

The strengths of this study were the long-term observation for a minimum time of 10 years, the performance of the same surgical technique of TKA by a single team, and avoidance of bias by separately enrolling surgeons and evaluators. Moreover, the validity of the radiological assessment was properly determined and reliable data were collected.

However, this study has certain limitations. First, the study was of a single-arm design without a control group. Second, outcomes were not assessed beyond 10 years of follow-up. Therefore, further studies are warranted to confirm our findings.

## Conclusions

This study demonstrated that the current model of alumina medial pivot TKA yielded good clinical results and survivorship over a minimum 10-year follow-up period. Medial pivot TKA made from alumina ceramic can be safely used in clinical practice.

## Data Availability

The datasets generated and/or analyzed during the current study are not publicly available due to institutional policy but are available from the corresponding author upon reasonable request.

## Abbreviations

TKA: Total knee arthroplasty
CoCr: Cobalt-chrome
PE: Polyethylene
RLL: Radiolucent line
ML/AP: Mediolateral/anteroposterior
ROM: Range of motion
KSS: Knee society score
SD: Standard deviation
